# Comparing Competitive Fitness of West Nile Virus Strains in Avian and Mosquito Hosts

**DOI:** 10.1371/journal.pone.0125668

**Published:** 2015-05-12

**Authors:** Gabriella Worwa, Sarah S. Wheeler, Aaron C. Brault, William K. Reisen

**Affiliations:** 1 Center for Vectorborne Diseases, Department of Pathology, Microbiology and Immunology, School of Veterinary Medicine, University of California, Davis, California, United States of America; 2 Division of Vector-Borne Diseases, Centers for Disease Control and Prevention, Fort Collins, Colorado, United States of America

## Abstract

Enzootic transmission of West Nile virus (WNV; *Flaviviridae*, *Flavivirus*) involves various species of birds and ornithophilic mosquitoes. Single nucleotide substitutions in the WNV genome may impact viral fitness necessary for WNV adaptation and evolution as previously shown for the WN02 genotype. In an effort to study phenotypic change, we developed an *in vivo* fitness competition model in two biologically relevant hosts for WNV. The House Finch (HOFI; *Haemorhous mexicanus*) and *Culex tarsalis* mosquitoes represent moderately susceptible hosts for WNV, are highly abundant in Western North America and frequently are infected with WNV in nature. Herein, we inoculated HOFIs and *Cx*. *tarsalis* competitively (dually) and singly with infectious-clone derived viruses of the founding California isolate COAV997-2003 (COAV997-IC), the founding North American isolate NY99 (NY99-IC), and a 2004 field isolate from California (CA-04), and compared the replicative capacities (fitness) of these viruses to a genetically marked virus of COAV997 (COAV997-5nt) by measuring RNA copy numbers. COAV997 and COAV997-5nt exhibited neutral fitness in HOFIs and *Cx*. *tarsalis*, and the temperature-sensitive phenotype of COAV997 did not affect replication in HOFIs as none of the infected birds became febrile. The NY99 and CA-04 isolates demonstrated elevated fitness in HOFIs compared to COAV997-5nt, whereas all viruses replicated to similar titers and RNA copies in *Cx*. *tarsalis*, and the only fitness differences were related to infection rates. Our data demonstrated that competitive replication allows for the sensitive comparison of fitness differences among two genetically closely related viruses using relevant hosts of WNV while eliminating host-to-host differences. In conclusion, our approach may be helpful in understanding the extent of phenotypic change in fitness associated with genetic changes in WNV.

## Introduction

West Nile virus (WNV, *Flaviviridae*, *Flavivirus*) is an enveloped single-stranded, positive-sense RNA virus that is maintained in an enzootic transmission cycle between birds within the order *Passeriformes* and ornithophilic *Culex* mosquitoes. Historically, WNV strains belonging to the founding East Coast genotype circulated in the Eastern United States during 1999–2002 [1]. Within five years, WNV dispersed westward and a new North American dominant genotype (WN02) emerged leading to the complete displacement of NY99 by 2004 [2, 3]. The first reported introduction of WN02 into California occurred in Imperial County during the summer of 2003 when infectious virus (COAV997-2003) was isolated from a RT-PCR-positive *Cx*. *tarsalis* mosquito pool [4]. In subsequent years WNV has been detected throughout California, indicating that the virus had become endemic and was causing recurrent outbreaks [4–7].

Three single nucleotide changes in the E and NS3 genes of the WN02 consensus sequence were identified compared to the NY99 genome [2, 8], and these mutations were correlated with increased vector infectivity facilitating the displacement of NY99. Vector competence studies in *Cx*. *tarsalis* and *Culex pipiens* revealed that WN02 showed a shorter extrinsic incubation period leading to transmission two to four days earlier than NY99 [8, 9], and WN02 replication was even more efficient when infected *Cx*. *pipiens* were held at warm temperatures [10]. Interestingly, WN02 and NY99 did not exhibit fitness differences *in vitro* when assessed in chicken embryo fibroblast (DF-1), African Green Monkey kidney (Vero) and *Aedes albopictus*-derived (C6/36) cells [8, 9, 11].

Isolates of the founding East Coast genotype typically have been associated with high virulence and mortality among American Crows (AMCR; *Corvus brachyrhynchos*) and other corvids [12, 13]. A single T249P substitution in the helicase protein has been demonstrated to confer increased virulence in AMCRs [13] contributing to the widespread outbreak in 1999 [1]. This helicase mutation has been positively selected and retained in WN02 genotype strains [14].

Sequencing of the founding California COAV997-2003 isolate (WN02 genotype) revealed mutations in the NS1 and NS4A genes that were associated with a temperature sensitive phenotype in duck embryonic fibroblast (DEF) cells at 44°C, suggesting that replication of this isolate might be impeded in febrile avian hosts [11]. A previous study demonstrated that differential growth kinetics of NY99 and an attenuated Kenyan WNV strain in DEF cells were comparable to viremia profiles obtained from infected AMCRs [15].

Alternate replication within the vertebrate host and insect vector presumably limits genetic variability among arthropod-borne viruses [16, 17]. However, once positively selected, these genetic changes may affect viral fitness in one host, but may not necessarily translate to improved fitness in the alternate host [18]. In addition, species-specific susceptibility to WNV among birds and mosquitoes also influences the replication of WNV [12, 19]. Corvids, such as Yellow-billed Magpies (*Pica nuttalli*), Western Scrub-Jays (*Aphelocoma californica*) and AMCRs, are considered highly competent species, developing peak viremia titers over 10 log_10_ PFU/mL, whereas moderately susceptible House Sparrows (HOSP; *Passer domesticus*) and House Finches (HOFI; *Haemorhous mexicanus*) usually develop viremias in the 6 to 8 log_10_ PFU/mL range, and doves, pigeons, quail and chickens exhibit low susceptibly with peak titers usually less than 4 log_10_ PFU/mL [12, 19]. Elevated virulence is a hallmark of WNV fitness in the avian host and typically is characterized by high viremia [13] and sometimes accompanied by high fever of up to 45°C [15]. In animals that succumb to infection, viremia and fever often persist while the onset of neutralizing antibodies remains delayed or inadequate for protection [20].

*Culex* mosquitoes are competent vectors of WNV, but the median infectious dose is species-dependent [19]. Moderately competent *Cx*. *tarsalis* require a median infectious dose that corresponds well with peak viremias of HOFIs and HOSPs [21]. *Culex tarsalis* from California infected orally with 7 log_10_ PFU/mL of WNV developed infection and transmission rates of 74 to 94% and 10 to 60%, respectively, after incubation at 28°C for 14 days [22]. Fitness of WNV in the vector is characterized by dose-dependent susceptibility to infection, with initial replication of WNV in the midgut cells, dissemination into the hemocoel, and subsequent infection and replication within salivary gland cells, leading to WNV transmission through salivation during host blood feeding. In addition to competence, host-feeding patterns of mosquitoes determine exposure to avian species and, therefore, WNV viremias [23].

Clearly, even single nucleotide changes in the WNV genome may give rise to new genotypes and significantly impact the fitness and virulence of WNV in avian and mosquito hosts. Although spatio-temporal evolution can be tracked by sequencing of highly variable portions of the genome such as the prM/E region [2], certain mutations may not necessarily relate to fitness and virulence observed *in vivo*, thereby limiting the understanding on how emerging WNV genotypes impact transmission dynamics. Importantly, genetic changes leading to subsequent fitness differences may not always be detectable *in vitro* [8, 9]. This emphasizes the need for *in vivo* models which provide a meaningful system to assess the fitness phenotypes of genetically related strains of WNV.

Herein, we describe in detail an *in vivo* fitness competition model in two moderately susceptible hosts for WNV, HOFI (*Haemorhous mexicanus*) and *Cx*. *tarsalis* mosquitoes, and show that this model accurately depicts the viral capacity of two viruses replicating concurrently in the same host. Concurrent replication results in competition, allowing for the direct and sensitive detection of minimal fitness differences between two viruses, while eliminating inter-host variability. Competition studies, therefore, have a clear advantage over independent, side-by-side competence studies and may reduce the number of animals required to detect statistically significant phenotypic differences.

Using an infectious clone-derived virus (COAV997-IC) of the founding California COAV997-2003 isolate [11], we previously generated the genetically marked COAV997-5nt mutant utilized in the current *in vivo* fitness competition study [24]. The COAV997-5nt contains five nucleotide substitutions (CTCTCC → TTGAGT) in the envelope gene at nucleotide positions 2449 and 2451–2454 [24]. Both COAV997-IC and COAV997-5nt demonstrated indistinguishable growth profiles in Vero and C6/36 cells over the course of three passages and yielded similar RNA copy numbers indicating fitness neutrality *in vitro* [24]. A quantitative RT-PCR (qRT-PCR) assay using specific reverse primers was developed to distinguish between RNA from wildtype COAV997-IC and COAV997-5nt in mixed competition experiments [24]. This assay showed a linear dynamic detection range of at least 6 log_10_ RNA copies with almost identical amplification efficiencies between wildtype and COAV997-5nt and a calculated detection limit of 250 RNA copies [24].

In the current study, we used the aforementioned qRT-PCR assay and the *in vivo* fitness competition model in HOFIs and *Cx*. *tarsalis* to demonstrate fitness neutrality between COAV997-IC and COAV997-5nt for both hosts. Additionally, we competed a NY99 genotype strain and a 2004 California WN02 genotype isolate against COAV997-5nt that revealed increased fitness for HOFIs but not *Cx*. *tarsalis* compared to COAV997-5nt. In summary, we present a sophisticated *in vivo* model to evaluate WNV phenotypes and quantitatively discern fitness differences among closely related WNV strains. This system will be helpful in tracking the spatio-temporal phenotypic evolution of WNV and may help to determine the epidemic potential of certain emerging genotypes.

## Results and Discussion

### Titer and RNA copies confirm similar doses of wildtype viruses and COAV997-5nt in bird and mosquito inocula

Viral titers in HOFI inocula were determined by plaque assay titration and ranged between 4.1–4.4 log_10_ PFU/mL with little deviation among samples (Table 1). Similarly, titers in blood meals and five fully engorged mosquitoes collected after blood feeding showed matching titers among groups with 6.3–6.8 log_10_ PFU/mL in blood meals and 4.5–4.7 log_10_ PFU/mL in mosquitoes (Table 2). These titers represent biologically relevant doses typically found during natural WNV transmission between HOFIs and *Cx*. *tarsalis* [21]. Analysis by qRT-PCR amplicons revealed slightly higher COAV997-5nt RNA copy numbers compared to the wildtype viruses (COAV997-IC, NY99-IC, CA-04) for all inocula. Despite equal viral titers, we previously noted increased COAV997-5nt RNA copies after *in vitro* replication [24]. Therefore results from final samples were normalized to the initial dose using the calculated RNA ratios between wildtype and COAV997-5nt at the time of inoculation as determined in Tables 1 and 2.

**Table 1:** Viral titer and RNA copy numbers from HOFI inocula and relative RNA input ratios between groups.

Group	Birds (n)	Titer (log_10_ PFU/mL)^a^	RNA copy wildtype^a^	RNA copy COAV997-5nt^a^	RNA ratio
COAV997-IC + COAV997-5nt	6	4.18 ± 0.02	87 ± 45	454 ± 170	0.19^**b**^
NY99-IC + COAV997-5nt	6	4.16 ± 0.03	75 ± 21	480 ± 8	0.15^**b**^
CA-04 + COAV997-5nt	6	4.07 ± 0.01	151 ± 50	376 ± 65	0.40^**b**^
COAV997-IC	6	4.12 ± 0.26	244 ± 150	0	0.22^**c**^
COAV997-5nt	6	4.19 ± 0.16	0	1070 ± 133	n/a
NY99-IC	6	4.14 ± 0.04	121 ± 63	0	0.11^**c**^
CA-04	6	4.36 ± 0.08	252 ± 41	0	0.23^**c**^
Mock-inoculated	5	0	0	0	n/a

^a^ Mean value and standard deviation calculated from duplicates of each inoculum.

^b^ Relative input RNA ratio between competing wildtype virus and COAV997-5nt calculated from mixed infection groups.

^c^ Relative input RNA ratio between wildtype virus and COAV997-5nt calculated from singly infected groups. Each ratio accounts for differences between the inoculum of either COAV997-IC, NY99-IC or CA-04 groups compared to the COAV997-5nt group.

Experimental groups and the total number (n) of infected birds per group are shown on the left. Viral titers of HOFI inocula were determined by plaque assay titration and are expressed as log_10_ PFU/mL. Titrations were performed in duplicate utilizing freshly prepared inocula and remaining inocula saved from syringes after bird injections. RNA copy numbers were determined in duplicate for wildtype viruses and COAV997-5nt by allele-specific qRT-PCR. The relative input (inoculum) ratio between RNA copies from wildtype virus and COAV997-5nt was calculated for each mixed competition group by dividing the wildtype RNA copies by COAV997-5nt RNA copies. The same ratio was calculated for singly infected groups in reference to the singly infected COAV997-5nt inoculum. Titers and RNA copies are followed by respective standard deviations resulting from duplicate values.

**Table 2:** Viral titer and RNA copy numbers from Culex tarsalis blood meals and relative input ratios between groups.

Group	Collected females (n)	Blood meal titer (log_10_ PFU/mL)	Mosquito titer (log_10_ PFU/mL)^a^	Mosquito RNA copy wildtype^a^	Mosquito RNA copy COAV997-5nt^a^	RNAratio
COAV997-IC + COAV997-5nt	30	6.58 ± 0.16	4.68 ± 0.23	13,047 ± 3,697	65,592 ± 9,825	0.19^b^
NY99-IC + COAV997-5nt	30	6.25 ± 0.35	4.62 ± 0.17	7,337 ± 2,236	58,762 ± 15,416	0.12^b^
CA-04 + COAV997-5nt	30	6.44 ± 0.05	4.51 ± 0.09	10,652 ± 3,339	52,811 ± 7,530	0.20^b^
COAV997-IC	30	6.48 ± 0.25	4.62 ± 0.13	16,296 ± 3,368	0	0.13^c^
COAV997-5nt	29	6.58 ± 0.03	4.57 ± 0.14	0	124,380 ± 24,324	n/a
NY99-IC	30	6.80 ± 0.14	4.57 ± 0.21	10,692 ± 7,144	0	0.08^c^
CA-04	30	6.53 ± 0.32	4.55 ± 0.19	10,856 ± 6,547	0	0.08^c^
Mock-inoculated	30	0	0	0	0	n/a

^a^ Mean value and standard deviation of five fully engorged females frozen immediately after blood meal uptake.

^b^ Relative input RNA ratio between competing wildtype virus and COAV997-5nt calculated from mixed infection groups.

^c^ Relative input RNA ratio between wildtype virus and COAV997-5nt calculated from singly infected groups. Each ratio accounts for differences between the inoculum of either COAV997-IC NY99-IC or CA-04 groups compared to the COAV997-5nt group.

Experimental groups and the total number (n) of living females collected on day 14 are presented on the left. Viral titers of the infectious blood meal and of five fully engorged females collected right after blood meal exposure of each group were analyzed by plaque assay titration and are shown as log_10_ PFU/mL with standard deviation. The relative input (inoculum) ratio between the mean RNA copies from wildtype viruses and COAV997-5nt in the five mosquitoes was calculated for each mixed competition group by dividing the wildtype RNA copies by COAV997-5nt RNA copies followed by standard deviation. The same ratio was calculated for wildtype singly infected mosquitoes in reference to the COAV997-5nt singly inoculated mosquitoes.

### HOFIs do not develop fever during acute viremia

**Fig 1:**
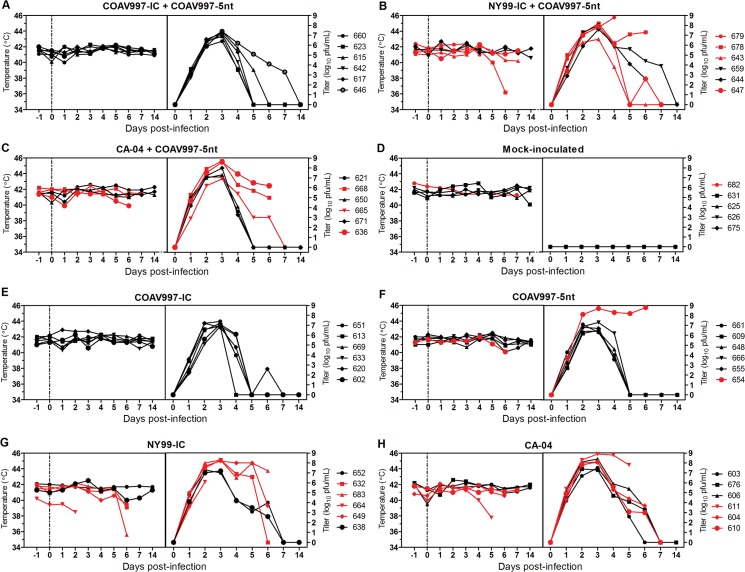
Body temperatures and viremia in HOFIs. Cloacal body temperatures in °Celsius are presented on the left y-axis and viral titers in serum on the right y-axis for each group (A-H) with days post infection (dpi) on both x-axes. Cloacal body temperatures were recorded daily starting one day prior inoculation until 7 dpi and again on 14 dpi. Day 0 and the dotted line represent the day of inoculation. A total of 100 μL of blood was obtained daily from jugular venipuncture between 1 and 7 dpi and on 14 dpi and mixed in DMEM diluent to obtain a serum dilution of 1:10 after centrifugation. Viral titers were determined by plaque assay titration and are presented as log_10_ PFU/mL after accounting for the 1:10 dilution. Red lines indicate HOFIs that died before the end of project day (14 dpi) and these animals were also used to calculate mortality in Fig 4.

Day 0 temperature reads from all birds averaged 41.3°C and served as the baseline temperature for this study. Surprisingly, none of the infected birds exhibited fever during peak viremia (Table 3) between 2–4 dpi, with cloacal body temperatures not differing significantly from the baseline temperature on any day (Fig 1). In fact, elevated viremia was accompanied by hypothermia during terminal stages of disease in HOFIs 678, 636, 683, 664 and 611, but not in surviving infected birds or in mock-inoculated birds (Fig 1). These 5 terminal birds presented a “puffed-up” plumage appearance, most likely in an attempt to upregulate their body temperatures. This observation contrasts studies in corvids that reported fevers up to 45°C [15], thereby potentially impairing replication of temperature-sensitive strains of WNV [11, 15]. In addition to a host-specific response, factors secondary to acute WNV infection such as starvation and loss in body condition also may be associated with hypothermia.

**Table 3:** Peak viremia and presence of neutralizing anti-WNV antibodies (nAb) in sera of HOFIs before infection and on day of death.

Group	HOFI #	nAb before infection	nAb at death	Day of death	Peak viremia (log_10_ PFU/mL)
COAV997-IC + COAV997-5nt	660	neg.	pos.	14	6.4
	623	neg.	pos.	14	6.9
	615	neg.	neg.	14	7.4
	642	neg.	pos.	14	7.1
	617	neg.	pos.	14	7.2
	646	neg.	pos.	14	7.4
NY99-IC + COAV997-5nt	679	neg.	neg.	5	8.8
	678	neg.	pos.	7	8.2
	643	neg.	pos.	9	6.6
	659	neg.	neg.	14	8.0
	644	neg.	pos.	14	7.6
	647	neg.	pos.	10	7.9
CA-04 + COAV997-5nt	621	neg.	pos.	14	7.1
	668	neg.	pos.	7	8.7
	650	neg.	pos.	14	7.3
	665	neg.	pos.	12	6.9
	671	neg.	pos.	14	8.0
	636	neg.	pos.	7	8.6
COAV997-IC	651	neg.	neg.	14	7.4
	613	neg.	neg.	14	6.9
	669	neg.	neg.	14	7.2
	633	neg.	pos.	14	6.9
	620	neg.	pos.	14	6.8
	602	neg.	neg.	14	7.1
COAV997-5nt	661	neg.	pos.	14	6.3
	609	neg.	pos.	14	6.5
	648	neg.	pos.	14	6.4
	666	neg.	pos.	14	7.3
	655	neg.	pos.	14	6.7
	654	neg.	pos.	7	8.7
NY99-IC	652	neg.	pos.	14	7.0
	632	neg.	pos.	7	8.3
	683	neg.	pos.	6	8.3
	664	neg.	neg.	3	6.1
	649	neg.	pos.	6	8.2
	638	neg.	pos.	14	7.2
CA-04	603	neg.	pos.	14	7.5
	676	neg.	neg.	14	7.3
	606	neg.	pos.	14	8.4
	611	neg.	pos.	6	8.9
	604	neg.	pos.	7	8.1
	610	neg.	pos.	14	8.1
Mock-inoculated	682	neg.	neg.	11	0
	631	pos.	pos.	14	0
	625	neg.	neg.	14	0
	626	neg.	neg.	14	0
	675	neg.	neg.	14	0

All HOFI sera were tested for nAb at a 1:20 dilution before infection and at the time of death with the end of the project at 14 dpi. Neutralization of >90% of 50–90 PFU was considered positive using a plaque reduction neutralization assay (PRNT_90_). Peak viremia titers determined by plaque assay titration are shown as log_10_ PFU/mL and occurred on 3 dpi or 4 dpi.

### COAV997-IC does not exhibit impaired replication *in vivo*

Peak viremia was detected at 3 dpi for all HOFIs except for 679 (4 dpi) and 664 (2 dpi) (Table 3). Birds infected singly with COAV997-IC or COAV997-5nt developed peak viremias ranging 6.8–8.7 log_10_ PFU/mL (Table 3, Fig 1), demonstrating that replication was not impaired *in vivo* in the absence of fever and was potentially sufficient to infect *Culex* mosquitoes. In contrast, growth of COAV997-IC was greatly reduced at 44°C in duck embryonic fibroblast (DEF) cells, suggesting attenuation in febrile avian hosts [11]. Cell culture conditions may not mimic all aspects of viral replication in avian and vector hosts and that highlights the importance of relevant *in vivo* models for phenotypic characterization of WNV and other arboviruses [25].

### COAV997-IC and COAV997-5nt are equally fit in HOFIs

**Fig 2:**
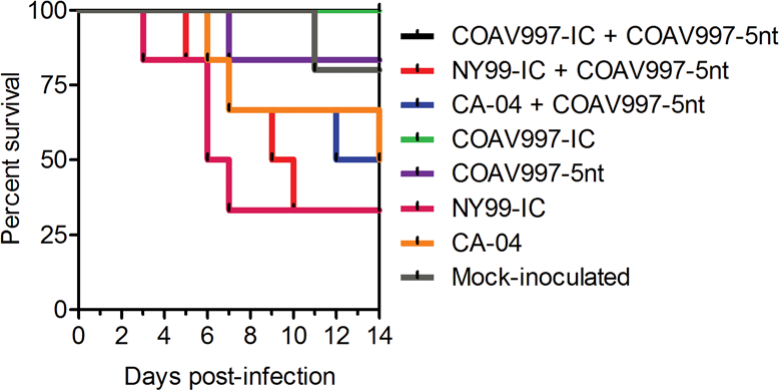
HOFI mortality rates. The percent survival of HOFIs (y-axis) is shown for each experimental group over the course of 14 dpi (x-axis). The lower the survival indicated by percent decrease, the higher the mortality rate in that group.

**Fig 3:**
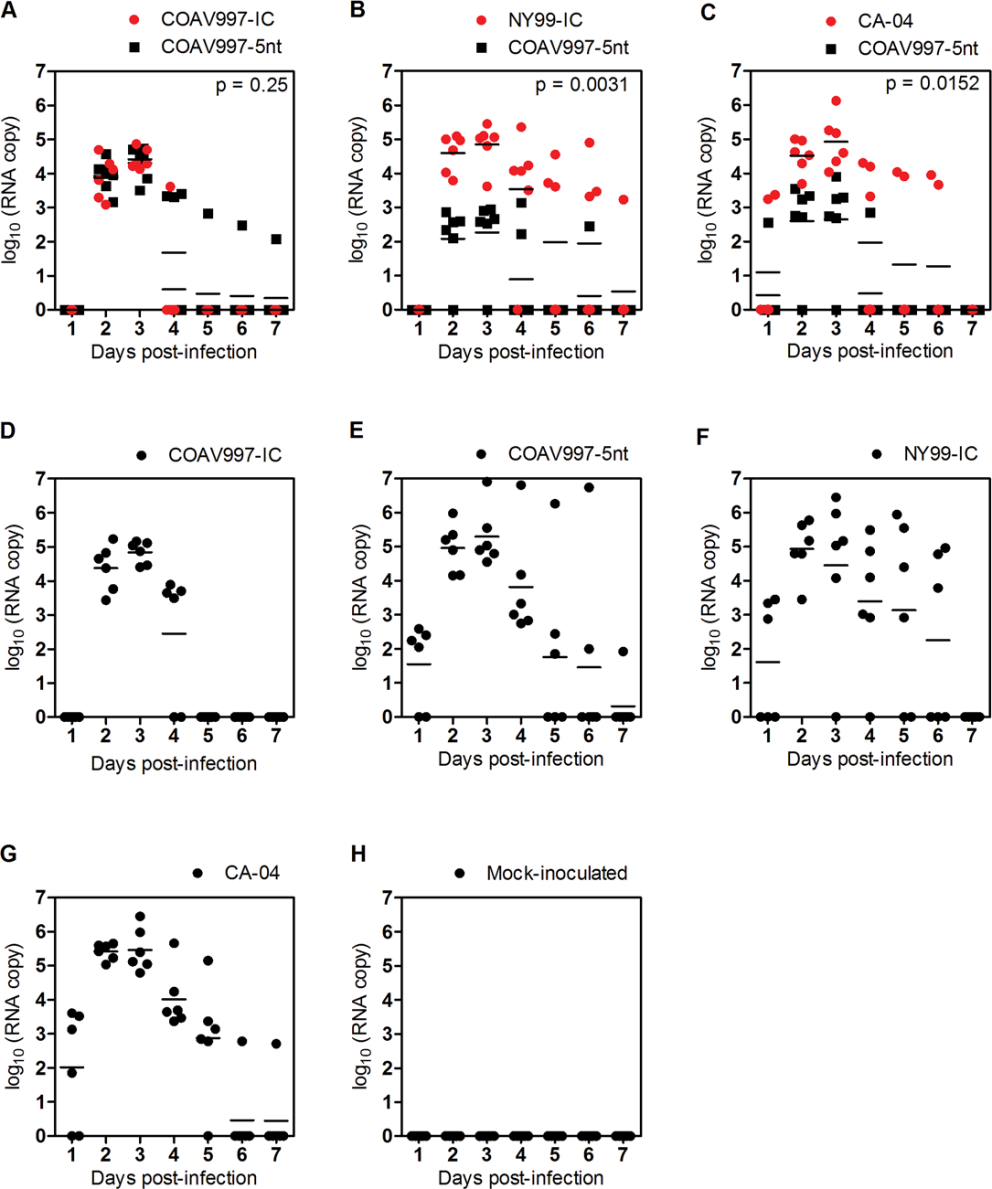
RNA copy numbers in serum of HOFIs. Panels A-H present RNA copies as log_10_ on the y-axis and the result for each bird is presented by a dot as a function of time (days post-infection on x-axis) for all groups. RNA copy numbers from sera were determined by allele-specific qRT-PCR for dually and singly infected animals. Samples taken at 14 dpi tested negative and were therefore not included. In plots representing viral competitions (A-C) red color indicates RNA detected for wildtype viruses (COAV997-IC, NY99-IC, CA-04) and black color represents COAV997-5nt detected RNA. Horizontal lines between dots indicate the mean RNA copy number per day and p values below 0.05 provide statistical significance of differences between wildtype and COAV997-5nt RNA copy numbers.

Infection with COAV997-IC and COAV997-5nt was followed by death in 0 of 6 and 1 of 6 birds, respectively, indicating comparable low mortality in HOFIs (Figs 1 and 2). Using a plaque assay in Vero cells [26], we determined serum viral titers (Fig 1) and compared them utilizing a repeated measures two-way ANOVA of variance. Serum titers of HOFIs singly infected with either COAV997-IC or COAV997-5nt were not significantly different (p>0.05), and there were also no titer differences between the competitively infected group (COAV997-IC + COAV997-5nt) and the singly infected COAV997-IC and COAV997-5nt groups (p>0.05). Next, we quantified RNA copies of COAV997-IC and COAV997-5nt by qRT-PCR in the sera of competitively infected HOFIs (Fig 3A) and found no significant differences (p = 0.25) at any time points between 1–7 dpi. When comparing COAV997-IC RNA copies from singly and dually infected birds, we found significant differences on 4 dpi, but not on any other days. This also was found for COAV997-5nt from singly and dually infected birds on 4 dpi, indicating increased variability when making comparisons among different groups. In summary, COAV997-IC and COAV997-5nt replicated to equal viral titers and RNA copy numbers during viremia in dually infected HOFIs indicating fitness neutrality *in vivo*.

### NY99-IC and CA-04 demonstrate increased replicative fitness

Identical mortality rates of 67% and 50% were observed for N99-IC and CA-04, respectively, in both competitively and singly infected groups (n = 6), suggesting that the specific virulence of NY99 and CA-04 may be reproduced in the presence of the less virulent COAV997-5nt (Figs 1 and 2). The same NY99-IC strain previously caused similar mortality of 63% in HOFIs [19]. NY99-IC showed increased fitness with RNA copy numbers being significantly greater (p = 0.003) compared to COAV997-5nt (Fig 3B). Similarly, CA-04 replicated to higher RNA copies (p = 0.01) than COAV997-5nt (Fig 3C). There were no differences (p>0.05) in RNA copies of NY99-IC and CA-04 when comparing the results from competitively and singly infected birds.

### Inefficient antibody production following COAV997-IC infection

Plaque reduction neutralization titers (PRNT) were determined in end point sera when the experiment was terminated on 14 dpi or on the last available sample before death (Table 3). All sera were tested at a 1:20 dilution and were considered positive when 90% of 50–100 PFU were neutralized (PRNT_90_). From a total of 28 HOFIs euthanized on 14 dpi, 21 showed detectable neutralizing antibodies (nAb), whereas serum from seven birds did not neutralize 90% of 50–100 PFU of WNV. Four of those seven birds were infected singly with COAV997-IC, potentially indicating that the immune response might be slow or low after COAV997 infection compared to other strains including COAV997-5nt, despite the fact that these birds developed peak viremias of 6.9–7.4 log_10_ PFU/mL (Table 3, Fig 1E); other negative birds were infected with COAV997-IC + COAV997-5nt, NY99-IC + COAV997-5nt and CA-04 and had viremias >7 log_10_ PFU/mL.

### No evidence for tissue-specific fitness in HOFIs

**Fig 4:**
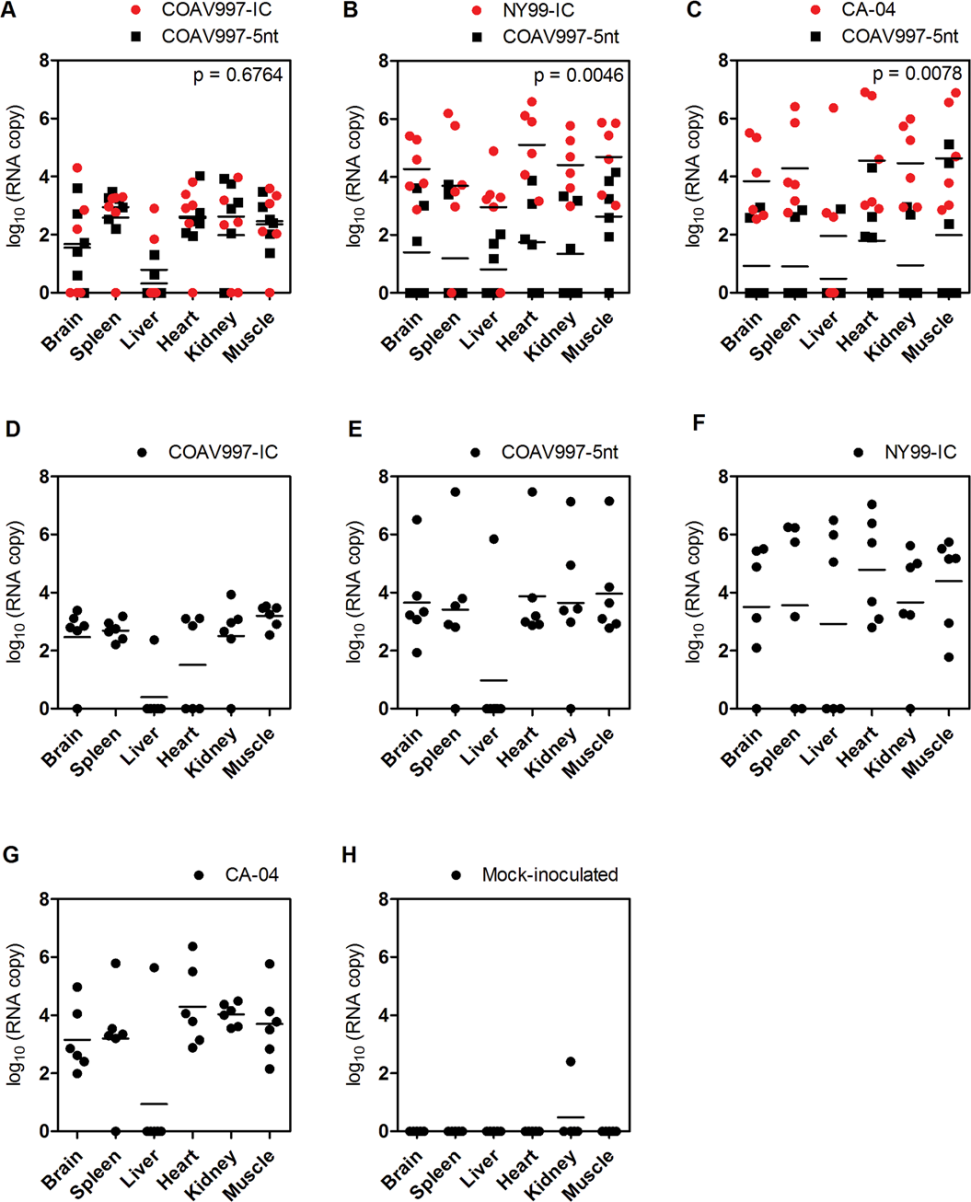
RNA copy numbers in tissues of HOFIs. Homogenates of brain, spleen, liver, heart, kidney and muscle were analyzed by qRT-PCR. RNA copies (as log_10_ on y-axis) for each bird and tissue (x-axis) are shown as dots in red and black color for wildtype and COAV997-5nt RNA, respectively, with horizontal lines indicating mean values. P values < 0.05 indicate statistically significant differences between wildtype and COAV997-5nt RNA copy numbers.

Individuals that succumbed during acute infection with detectable WNV titers in their sera (Fig 1) also showed higher RNA copy numbers in tissues compared to birds euthanized on 14 dpi (Fig 4). Tissues from birds infected competitively with COAV997-IC + COAV997-5nt (Fig 4A) did not show significant differences in the RNA copy numbers detected from the two viruses in any of the tissues (p = 0.67), providing more evidence that the two viruses have equal fitness in HOFIs. Similar to results obtained from sera, more RNA copies of NY99-IC (p = 0.004, Fig 4B) and CA-04 (p = 0.007, Fig 4C) were detected in the tissues of dually infected birds compared to COAV997-5nt. Therefore, the fitness outcome determined in multiple tissues at necropsy was not different compared to serum, indicating that there was no tissue-specific fitness observed for these strains (Figs 3 and 4).

### COAV997-IC and COAV997-5nt are equally fit in *Culex tarsalis*

**Fig 5:**
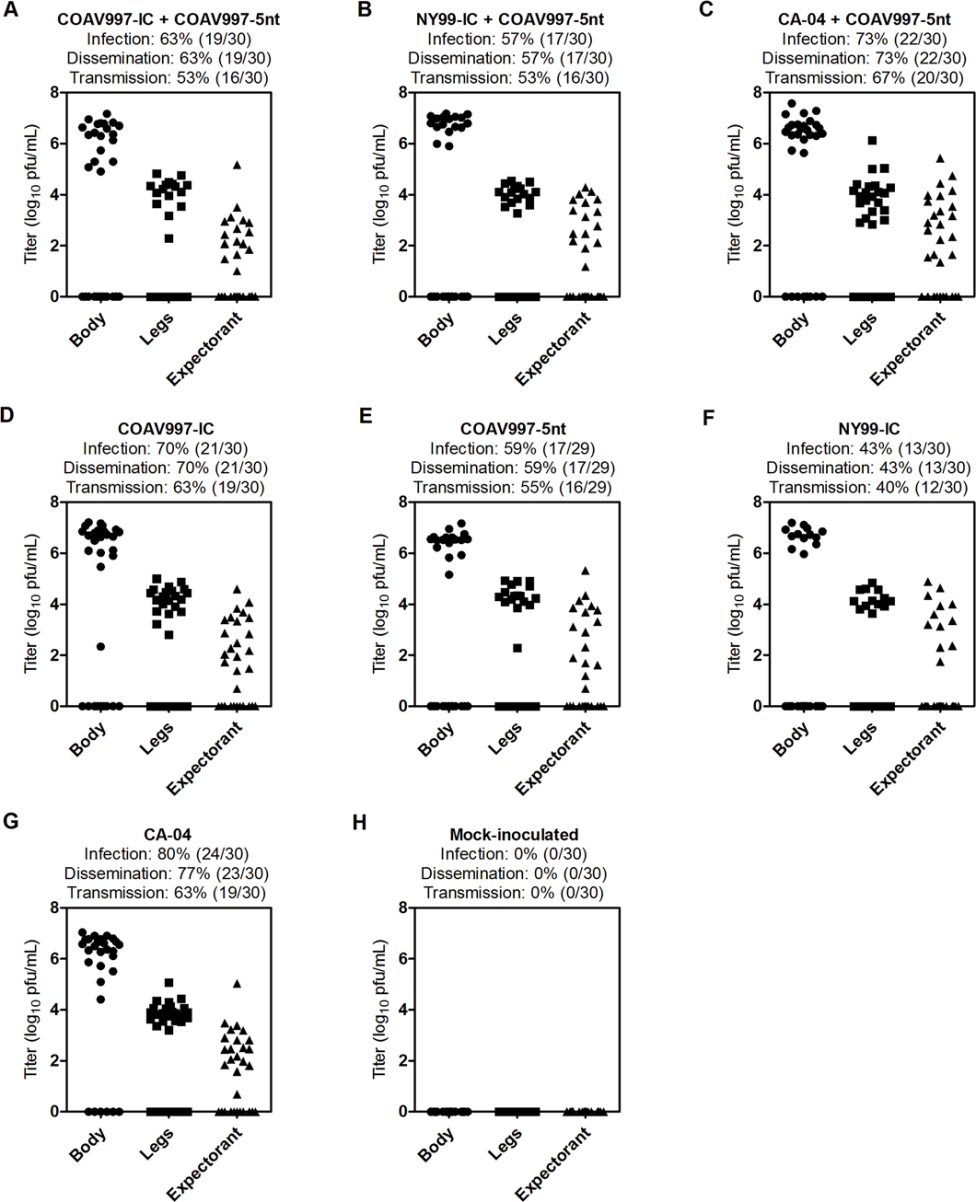
Viral titers in bodies, legs and expectorant of *Culex tarsalis*. Homogenates of mosquito bodies, legs and collected expectorant samples were serially diluted tenfold and analyzed by plaque assay titration. Results for each group are expressed as log_10_ PFU/mL in panels A-H and the calculated infection, dissemination and transmission rates are presented as percent (%) on the right with absolute numbers in parenthesis.

**Fig 6:**
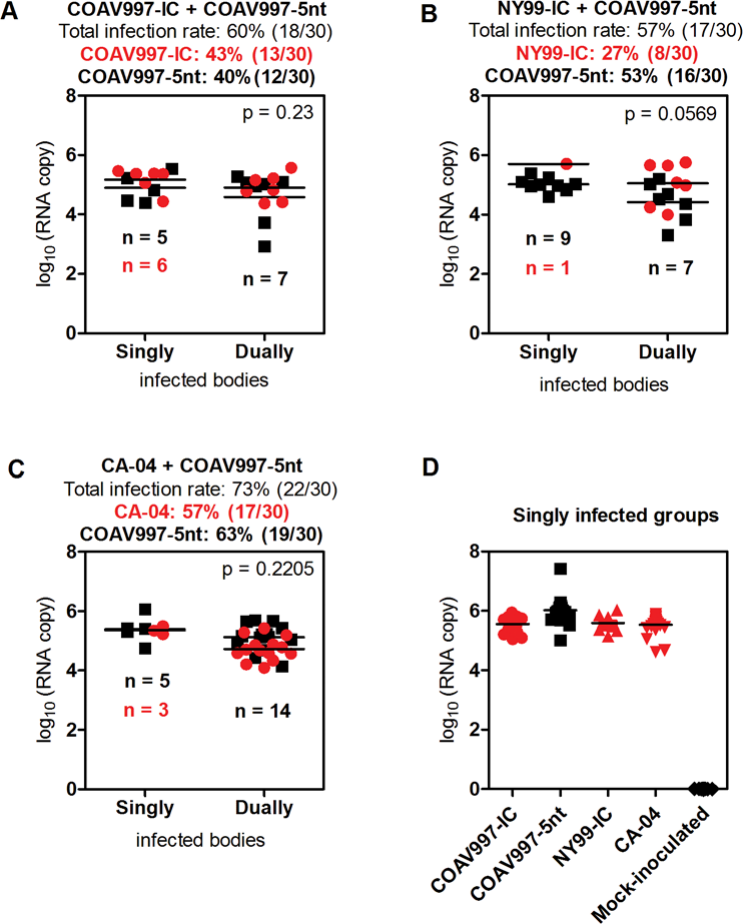
RNA copy numbers in *Culex tarsalis* bodies. RNA copy numbers as log_10_ were determined in mosquito bodies by qRT-PCR. Panels A-C present the RNA copy numbers from mixed competition groups and panel D from the four singly infected groups with wildtype RNA in red color and COAV997-5nt RNA in black color. In panels A-C results were divided into bodies that tested positive for one virus (singly infected) or both viruses (dually infected), and the respective specific infection rates are listed on the right. The total infection rates indicate how many mosquitoes were tested positive by either of the qRT-PCRs and therefore combine numbers from singly and dually infected mosquitoes. For singly infected bodies in panels A-D, each dot represents the RNA value for one body, whereas two dots are referring to two RNA values obtained from one dually infected body in panels A-C. Horizontal lines indicate the mean RNA copy value for each group. The number of RNA-positive bodies is indicated in parenthesis and the infection rates shown as percent (%).

**Fig 7:**
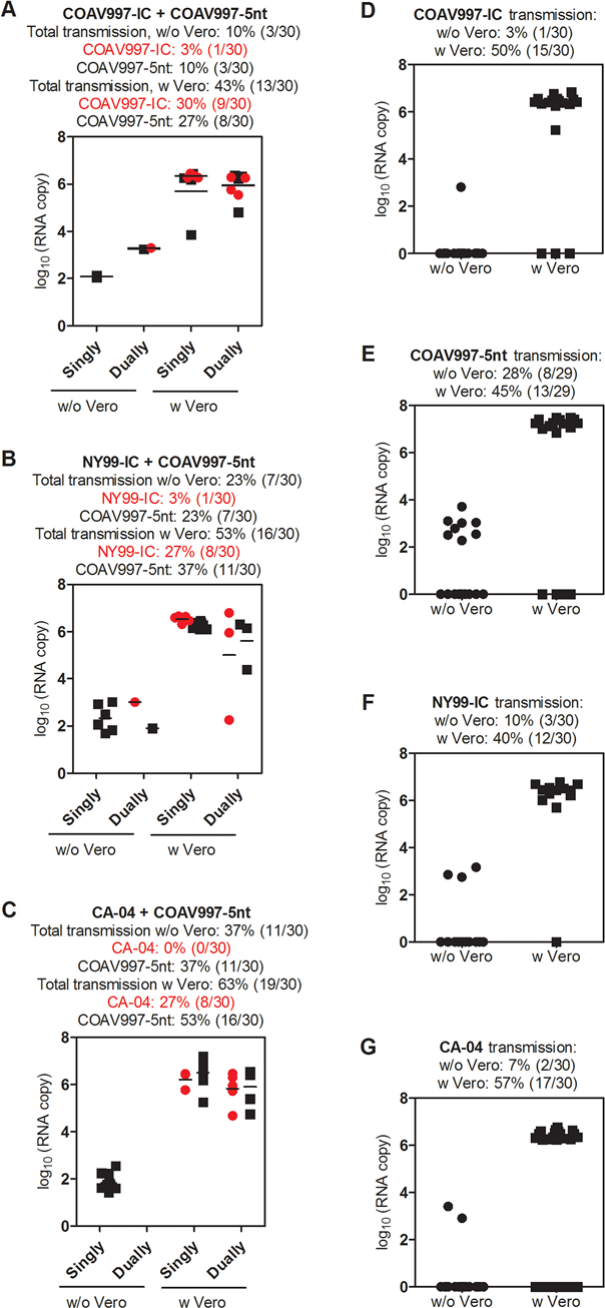
RNA copy numbers in *Culex tarsalis* expectorant before and after Vero cell passage. Mosquito expectorants from all groups were analyzed by qRT-PCR, but only few samples yielded detectable results. For that reason, we additionally passaged all samples once in Vero cells and analyzed supernatants by qRT-PCR to help visualize whether wildtype and COAV997-5nt infectious virus was present in the expectorant. For mixed competition groups (A-C) results were divided into singly and dually infected groups meaning that either one or both viruses were present in the sample. One dot is presented when one virus was detected (singly infected), whereas a black and red dot were assigned to one sample for wildtype and COAV997-5nt RNA, respectively, when both viruses were found in the sample. The total transmission rates for un-passaged and Vero cell passaged expectorants include all positives. In addition, virus-specific transmission rates were calculated individually from both singly and dually infected mosquitoes. Expectorants from singly infected mosquitoes (D-G) are presented as single dots and respective transmission rates are calculated before and after Vero cell passage.

Females were collected 14 days after blood feeding and the bodies, legs and expectorants were analyzed using plaque assay titration (Fig 5A) and qRT-PCR (Fig 6A). Viral titers obtained from bodies, legs and expectorants from competitively and singly COAV997-IC and COAV997-5nt infected mosquitoes showed no difference in magnitude (p>0.05). Rates of infection (63%, 70%, 59%), dissemination (63%, 70%, 59%) and transmission (53%, 63%, 55%) also were similar for COAV997-IC + COAV997-5nt, COAV997-IC and COAV997-5nt groups, respectively (Fig 5A, 5D and 5E). We then analyzed the bodies of competitively infected females by qRT-PCR and found that six bodies contained COAV997-IC RNA only, five contained COAV997-5nt RNA only, and seven bodies were positive for RNA from both viruses (Fig 6A). The quantity of RNA copies was not different (p = 0.23) between COAV997-IC and COAV997-5nt in mosquito bodies. When determining RNA copies in Vero cell passaged mosquito expectorant, we found 30% transmission for COAV997-IC and 27% transmission for COAV997-5nt (Fig 7A). Overall, this indicated that the genetically marked COAV997-5nt infected, replicated and was transmitted equally to the COAV997-IC virus in *Cx*. *tarsalis* (Fig 7A).

### Different infection, dissemination and transmission rates of NY99-IC and CA-04 despite similar titers in *Culex tarsalis*

Viral titers determined by plaque assay titration did not differ among bodies, legs and expectorants of mosquitoes from all groups collected at 14 dpi (p>0.05; Fig 5). However, NY99-IC showed 43% infection, 43% dissemination and 40% transmission, whereas CA-04 demonstrated 80% infection, 77% dissemination and 63% transmission as determined by plaque assay titrations of singly infected females (Fig 5F and 5G), indicating lower fitness of NY99-IC than CA-04 in *Cx*. *tarsalis*. Competitive infection of NY99-IC and CA-04 with the genetically marked COAV997-5nt virus resulted in rates that were similar to those averaged between singly infected groups (Fig 5B and 5C).

### NY99-IC shows reduced infection rate in *Culex tarsalis*, but not decreased RNA copy numbers compared to COAV997-5nt, COAV997-IC and CA-04

As described above, bodies, legs and expectorants yielded titers that were not statistically different (p>0.05; Fig 5), but the number of NY99-IC infected mosquitoes was lower than for the other viruses. We assessed the RNA copy number of NY99-IC and COAV997-5nt in bodies of dually infected females, and found that these were not quite significantly different (p = 0.056; Fig 6B). However, only one mosquito became singly infected with NY99-IC, whereas nine mosquitoes became infected singly with COAV997-5nt, and seven mosquitoes tested positive for both NY99-IC and COAV997-5nt when both viruses were presented in the blood meal (Fig 6B) leading to a calculated infection rate of 27% and 53%, respectively, in mixed infections. Overall, our data showed that on day 14 there were fewer *Cx*. *tarsalis* infected with NY99-IC compared to COAV997-5nt, but both viruses replicated to similar titers and RNA copy numbers.

### Bird isolate CA-04 is fit in *Culex tarsalis* and HOFIs

In the current study, the CA-04 virus, originally isolated from a dead Yellow-billed Magpie in 2004, exhibited high fitness and virulence in HOFIs (Figs 1C, 1H, 2, 3C, 3G and 4C, 4G). This isolate also demonstrated high fitness in *Cx*. *tarsalis* mosquitoes with infection rates of 80% determined by plaque assay titration of singly infected bodies (Fig 5G). When analyzing bodies after exposure to dual infection with COAV997-5nt, we found three CA-04 singly infected, five COAV997-5nt singly infected, and 14 dually infected mosquito bodies (Fig 6C), resulting in 57% infection rate for CA-04 and 63% infection for COAV997-5nt, with no significantly different RNA copy numbers between the two viruses (p = 0.22). We therefore did not observe a fitness trade-off effect for CA-04 as described previously for arboviruses during host alteration [16, 27].

### Less distinct differences in mosquito transmission rates among NY99-IC, CA-04 and COAV997-5nt compared to infection rates

All mosquito expectorants were analyzed by qRT-PCR before and after one passage in Vero cell culture (Fig 7) to assess fitness transmission differences. In NY99-IC singly infected mosquitoes, we detected virus-specific RNA in 40% of Vero cell passaged expectorants (Fig 7F). In contrast, 27% and 37% of Vero cell passaged expectorants from dually infected mosquitoes contained NY99-IC and COAV997-5nt RNA, respectively (Fig 7B). Vero cell propagated expectorant of CA-04 + COAV997-5nt yielded 27% transmission of CA-04 and 53% of COAV997-5nt (Fig 7C). In summary, the transmission rates calculated from Vero cell passaged expectorant using qRT-PCR agreed well with infectious virus detected by plaque assay titration for singly infected groups (Figs 5D–5G and 7D–7G). In dually infected groups, the combined transmission rates matched well with infectious virus detected by plaque assay, but the virus-specific transmission rates did not correspond well with virus-specific infection rates detected for NY99-IC and CA-04 (Figs 5B–5C and 7B–7C). This lack of concordance may be attributed to multiple aspects of vector competence including midgut infection, dissemination and transmission barriers. Also, decreased ability of NY99-IC to infect the midgut cells of *Cx*. *tarsalis* (infection rate) may be compensated by increased infection of the salivary gland cells leading to more efficient transmission (transmission rate) whereas the opposite may be true for CA-04.

### Significance and limitations of the *in vivo* fitness competition model

By utilizing competitive replication of genetically related WNV isolates in HOFIs and *Cx*. *tarsalis*, we were able to reproduce the phenotypic hallmarks of NY99-IC and two WN02 strains, COAV997-IC and CA-04. COAV997-IC and COAV997-5nt exhibited neutral fitness in birds and mosquitoes, indicating our genetic labelling did not alter fitness and validating our use of this founding strain in future competition studies. Although characterization of the fitness of NY99-IC and CA-04 was straight-forward in birds, interpretation of results from competitively infected mosquitoes proved to be more complex and will require the definition of fitness on more than one level. However, the ability to discern between singly and dually infected mosquitoes might provide valuable insights into the complexity of infection dynamics within vector mosquitoes. Limitations also included fitness being determined in only two moderately susceptible host species; however, this model may be expanded to include high and low competent hosts, because natural transmission dynamics involve rural and urban transmission cycles with a wide range of different avian and *Culex* species [6]. In the current study, we used day 14 to quantify mosquito infection and transmission, but collection of mosquitoes could be extended to multiple time points to interrogate infection and transmission dynamics during early and late competition.

### Concluding remarks

In the current study, competition in dually infected individuals facilitated the rapid screening for relative fitness differences between two viruses, while eliminating inter-host variability and reducing the number of animals. Small genetic changes in WNV can result in significant phenotypic changes and selected nucleotide substitutions may give rise to new and better adapted viruses. Our *in vivo* fitness competition model will help track the spatio-temporal phenotypic evolution of WNV in California compared to the founding COAV997-2003 isolate. It is unclear what facilitates WNV epidemics and whether outbreak isolates are associated with increased fitness in either host. The 2012 outbreak of WNV in Texas was associated with increased incidence in humans, but sequencing of isolates failed to demonstrate an association between genetic factors and outbreak magnitude [28]. Our model may help investigate whether outbreak isolates exhibit elevated fitness in birds and/or mosquitoes and may contribute to outbreak predictions based on the characterization of the viral fitness of emerging WNV strains.

## Materials and Methods

### Ethics

This study was performed in strict accordance with the recommendations in the Guide for the Care and Use of Laboratory Animals of the American Veterinary Medical Association (AVMA). The University of California is approved for animal experimentation under the National Institutes of Health (NIH) Animal Welfare assurance number A3433. Collection, care, maintenance and infection of House Finches was approved under the University of California Davis Institutional Animal Care and Use Committee (IACUC) approved protocol 15895, US Federal Fish and Wildlife permit MB082812-1, and the State Fish and Game collecting permit SC-002281. House Finches were trapped using modified Australian Crow traps operated by contractors at multiple vineyards near Bakersfield, CA under depredation permits which afforded us permission to remove birds for experimental purposes. No endangered species were caught when trapping wild House Finches. We made significant efforts to avoid death as an endpoint through daily observations of animals for the duration of 1–3 hours a day throughout the entire ABSL-3 phase of the experiment. During these observations, the health condition of each animal was assessed individually and within the cage group by a veterinarian (G. Worwa). Moribund animals (lethargic or unresponsive birds) and animals suffering from visible pain or distress were euthanized via inhalation of carbon dioxide as specified in IACUC protocol 15895. Death by carbon dioxide inhalation is listed as an acceptable euthanasia method for birds by the American Veterinary Medical Association (AVMA) in the AVMA Guidelines for Euthanasia of Animals 2013 Edition. However, death in House Finches due to acute West Nile virus infection often occurs very suddenly and therefore prevented a timely intervention for most birds in this study. Blood used to infect mosquitoes was collected from chickens maintained in accordance with IACUC protocol 15892. Chickens were purchased by the Kern Mosquito and Vector Control District in Bakersfield who gave permission for us to collect blood in accordance with procedures described and approved in IACUC protocol 15892. WNV strain COA977-2003 was isolated from *Cx*. *tarsalis* mosquitoes collected by dry-ice baited traps operated at the Wister State Wildlife Area in Imperial County, CA [33.277, 115.577] as part of the California Mosquito-Borne Encephalitis Virus Surveillance Program; specific collection permits were not required to operate mosquito traps. The biosafety level 3 facility was approved under Biological Use Authorization 0872 by the University of California, Davis, Environmental Health and Safety Institutional Biosafety Committee and USDA Permit 47901.

### Viruses

The COAV997-2003 isolate (NCBI GenBank accession no. JF703162) was made from a *Cx*. *tarsalis* mosquito pool collected in Imperial Valley, California, in July 2003 and represents historically the WN02-representative founding isolate from California [4]. Infectious-clone derived viruses of COAV997-2003 (COAV997-IC) and NY99 strain 382–99 (NY99-IC) were generated previously [11, 15], and the NY99-IC was a representative of the East Coast genotype. SAC-04-7168 (CA-04; NCBI GenBank accession no. DQ080059) was isolated from a Yellow-billed Magpie (*Pica nuttalli*) found dead in 2004 in Sacramento, California, one year after invasion of WNV into California [14]. The CA-04 isolate was passaged three times in Vero cells before use in this study. We previously described the construction of COAV997-5nt through substitution of five synonymous nucleotides (CTCTCC → TTGAGT) in the envelope gene of COAV997-IC at nucleotide positions 2449 and 2451–2454 [24]. Infectious clone derived viruses and COAV997-5nt were rescued initially from baby hamster kidney cells (BHK-21, clone 13, ATCC no. CCL-10) and subsequently grown in African green monkey kidney cells (Vero cells; obtained from the ATCC no. CCL-81) for stock production. The genotypes and phenotypes of the above viruses previously have been well characterized [11, 13, 24, 29]. We demonstrated similar replication dynamics and RNA copy yields for COAV997-IC and COAV997-5nt in Vero and C6/36 cells indicating that the two viruses are equally fit *in vitro* [24]. Although titers were almost identical, COAV997-5nt yielded slightly higher RNA copy numbers compared to COAV997-IC in Vero and C3/36 cells. Although these differences were not statistically significant [24], we accounted for this variation by normalizing all results to the actual RNA copy input at time of inoculation (Tables 1 and 2).

### Experimental design and inoculations

A total of eight experimental groups of HOFIs and *Cx*. *tarsalis* mosquitoes were used in this study: three competitively (dually) infected groups, four singly infected and one mock-infected group (Tables 1, 2 and 3). For the competitively infected groups the genetically marked COAV997-5nt was mixed 1:1 with COAV997-IC, NY99-IC or CA-04. The 1:1 mixture was achieved using equal numbers of plaque forming units (PFU) of each virus determined by plaque assay titration [26]. The COAV997-IC + COAV997-5nt group represented the group to be tested for neutral fitness *in vivo*, whereas NY99-IC + COAV997-5nt and CA-04 + COAV997-5nt groups were analyzed for differential fitness outcome in the *in vivo* model. Each of the four viruses was inoculated singly into birds and fed within an artificial blood meal to mosquitoes to compare results from single and dual infections. Mock-infection was performed using Dulbecco’s Modified Eagle Medium (DMEM; Life Technologies, Gibco, Carslbad, CA) without any additives and using the same sterile inoculation techniques as described below for each species.

Groups of six HOFIs were inoculated with a total of 1000 PFUs and five birds were mock-inoculated with DMEM by needle injection subcutaneously above the right pectoral muscle. This dose has been shown to be biologically relevant as it corresponded to the virus concentration expectorated by *Culex* mosquitoes [19, 30]. Inocula were prepared on ice by adjusting the titer of all viruses to 8 log_10_ PFU/mL and subsequent dilution to 4.3 log_10_ PFU/mL in DMEM. Birds infected with a single virus were inoculated with 50μL of the 4.3 log_10_ PFU/mL virus dilution. For animals infected dually with COAV997-5nt, a 1:1 mixture of the two viruses was made at 4.3 log_10_ PFU/mL of which 50μL were administered leading to a total calculated concentration of 500 PFU for each virus. Aliquots of all inocula were saved from the tubes in which the inoculum was prepared before inoculation and from the syringes used for injection after administration, and all samples were stored at -80°C until analysis. All inocula aliquots were analyzed by plaque assay titration [26] and qRT-PCR [24] to determine the titer and RNA copy number that was effectively used in the inocula (Table 1).

Mosquitoes were orally exposed to viruses using a virus-spiked infectious blood meal. (Heparinized blood was obtained from chickens held in a mosquito-proof aviary at the Arbovirus Field Station in Bakersfield and confirmed to be free from anti-WNV antibodies by plaque reduction neutralization assay (PRNT) [31]). First, the titer of all viruses was adjusted to 8 log_10_ PFU/mL in DMEM and then a final concentration of 7 log_10_ PFU/mL was obtained by an additional 1:10 dilution in heparinized chicken blood on ice. Mosquitoes were starved for 24 hours before being offered the infectious blood meal using a Hemotek membrane system (Discovery Workshops, Accrington, Lancashire, UK) for one hour in the dark at a blood meal temperature of 37C. Following blood meal exposure, mosquitoes were anesthetized with carbon dioxide and all engorged females were transferred into a fresh container. Aliquots of blood meals and five fully engorged females from each group were frozen at -80°C immediately after virus exposure and later analyzed by plaque assay titration and qRT-PCR to determine the titer and RNA copy numbers in the inocula (Table 2).

To account for relative differences in exposure to differential doses of competing viruses at the start of competition, we calculated a ratio of RNA input by dividing the RNA copy number from each of the wildtype viruses by the number of COAV997-5nt RNA copies per inoculum. Using this ratio, results were then normalized before analysis. This was also calculated for singly infected groups to facilitate side-by-side comparison of results (Tables 1 and 2).

### Data collection from House Finches

We selected the HOFI as a model avian host, because it is a moderately susceptible and a biologically relevant host of WNV that is abundant in North America and adapts easily to captivity and laboratory conditions. Using grain-baited traps, hatching-year HOFIs were collected in Kern County, CA during early summer 2010 and transferred to a mosquito-proof aviary at the Arbovirus Field Station in Bakersfield. All birds were administered 0.2mg/mL of chlorotetracycline (Fort Dodge, Overland Park, KS) in drinking water for 14 days. Serum from all birds was screened for antibodies against WNV, SLEV and WEEV with an immunosorbent assay [32] to exclude seropositive animals from the study. A total of 47 birds were transferred to the California Animal Health & Food Safety Laboratory (CAHFS) where they were housed in groups of six in individual cages within negative pressure HEPA-filtered Horsfall-Bauer units. Birds were allowed a two-week cage adaptation prior to infection during which absence of anti-WNV neutralizing antibodies (nAb) was confirmed in serum by PRNT_90_. All birds tested nAb-negative except for HOFI 631 (Table 3) which was moved to the mock-infected group prior to infection. HOFI 631 also showed detectable WNV RNA in the kidney homogenate (Fig 4H), suggesting that it was naturally infected with WNV but that viral RNA persisted after the viremia subsided before the animal was captured [33].

All animals were observed daily for clinical signs such as lethargy, ataxia, abnormal body posture, tremor and changes in plumage appearance. Cloacal body temperatures were recorded starting one day prior to inoculation and every day thereafter using a disinfected digital thermocouple (Fisher Scientific, USA) placed 1 cm into the cloaca prior to any other manipulations of the animal. During each blood draw, a total of 100 μL of blood was collected from each animal with a 28-gauge needle by jugular venipuncture between 1–7 days post infection (dpi) and upon termination of the study at 14 dpi. Blood was diluted in 450μL of DMEM containing 10% heat-inactivated fetal bovine serum (FBS; Invitrogen, USA), 100 U/mL penicillin, 0.1 g/mL streptomycin and 1.25 μg/mL amphotericin B (Life Technologies, Gibco, Carlsbad, CA) leading to a final approximate 1:10 dilution of serum. Birds were humanely euthanized by carbon dioxide inhalation and equally sized specimens of spleen, kidney, right pectorial muscle, liver, brain and heart were collected from all animals during necropsy. Sera and tissues were stored at -80°C until analysis by plaque assay titration, PRNT_90_ and qRT-PCR. On 11 dpi mock-infected HOFI 682 suffered a leg fracture and was humanely euthanized (Fig 2).

### Data collection from *Culex tarsalis* mosquitoes

A laboratory-adapted colony of *Cx*. *tarsalis* was used for all vector competition studies. Mosquitoes originated from the Kern National Wildlife Refuge in California and were reared under standard insectary conditions at 22°C and a photoperiod of 16:8 (L:D) hours at the Arbovirus Field Station in Bakersfield, CA. Mosquitoes were transferred to the biosafety level 3 insectary at the University of California in Davis prior infection.

After infectious blood meal exposure, engorged females were provided daily with a 10% sucrose solution and held for 14 days at 26C, 12:12 (L:D) hours and approximately 60% humidity. On day 14 all surviving females were counted (Table 2) and anesthetized using triethylamine (Fisher Scientific, USA). Expectorant was collected from each mosquito by placing the proboscis into a glass capillary tube filled with a 1:1 5% sucrose and 50% FBS solution. After approximately 10 minutes the proboscis was removed and content expelled by centrifugation into 250μL of DMEM containing 10% FBS, 100 U/mL penicillin, 0.1 g/mL streptomycin and 1.25 μg/mL amphotericin B (Life Technologies, Gibco, Carlsbad, CA). The legs were separated from the body using sterile technique and placed into cryovials containing two 5mm glass beads and 600μL of the above DMEM solution. Expectorant, legs and bodies were stored at -80°C until further processing.

The number of infectious virus particles in the mosquito expectorant was frequently below the qRT-PCR detection limit of 250 RNA copies (Fig 5) [24]. To maximize data on vector transmission as a key factor of WNV fitness, we passaged all expectorant samples once in Vero cells with subsequent quantification of RNA copies in addition to direct analysis of expectorant samples by qRT-PCR. Despite the fact that three rounds of replication in Vero cells previously did not affect the fitness of COAV997-IC and COAV997-5nt [24], we excluded any bias from cell culture passaging by using only qualitative data from passaged expectorant samples as to whether one, both or neither of the viruses were detected in the supernatant supporting the presence or absence of infectious virus particles in the original sample.

### Plaque assay titration, PRNT_90_ and RNA copy measurement by qRT-PCR

Bird tissues were added to 1mL and mosquito bodies and legs to 600μL of diluent and two 5 mm glass beads for homogenization on a Tissue Lyser (Qiagen, USA). Samples were triturated twice for two minutes at 24 Hz frequency and the homogenate clarified by centrifugation.

Infectious viral titers in bird sera and in mosquito bodies and expectorant samples were quantified by plaque assay titration [26]. Briefly, serially tenfold diluted samples were allowed to absorb on confluent Vero cell monolayers for 1 hour at 37°C and 5% CO_2_. An agarose-based overlay was added (nutrient medium, 0.5% agarose, 3% bicarbonate) and after 48 hours of incubation at 37°C and 5% CO_2_ a second overlay containing additional 3% neutral red was applied. The following day visible plaques were enumerated and the titer determined as log_10_ PFU/mL (Figs 1 and 5)

Sera taken prior to inoculation and all end point serum samples (14 dpi or last available serum sample before death) were tested by plaque reduction neutralization assay using a 90% neutralization cut-off (PRNT_90_) [31]. Briefly, all sera were inactivated at 56°C for 30 minutes and diluted 1:2 in virus solution containing 100 PFU (CA-04 isolate) making a final serum dilution of 1:20. After incubation of the serum-virus mixture for 1 hour at 37°C and 5% CO_2_, confluent Vero cell monolayers were inoculated and incubated for an additional hour at 37°C and 5% CO_2_. The same double-overlay system was used as described above for plaque assay titration. Sera producing a >90% reduction of 50–100 PFU were considered positive for nAbs (Table 3).

Viral RNA was extracted utilizing a MagMAX magnetic particle processor and MagMAX-96 Viral RNA isolation Kit (Applied Biosystems, USA) according to the manufacturer’s instructions. The allele-specific qRT-PCR for quantitative detection and distinction between COAV997-5nt and wildtype viruses was previously described [24]. Briefly, specific reverse primers WNV.TAQ.WT.2464.R and WNV.TAQ.5nt.2467.R-alt were designed to bind the wildtype sequence (CTCTCC) and COAV997-5nt (TTGAGT) at their 3’-ends, respectively. A total of 10uL viral RNA was mixed with 0.5 μL of WN.2417-2431.FAM (100 nM), 0.05 μL WNV.TAQ.2393.F (25 nM), 12.5 μL TaqMan One-Step RT-PCR Master Mix 2X (ABI, USA), 0.6 μL of TaqMan One-Step RT-PCR RNase Inhibitor, and 1.3 μL of DEPC-treated water. For specific wildtype and COAV997-5nt detection, 0.05 μL of either WNV.TAQ.WT.2464.R (25 nM) or WNV.TAQ.5nt.2467.R-alt were added to two separate master mixes. Samples and standards were run in duplicate with a final volume of 25 μL per reaction and amplified in 50 cycles in singleplex format concurrently on two 7900HT Fast Real-Time PCR Systems (ABI, USA). Quantification of RNA copies was accomplished using standard curves from plasmid-derived *in vitro* transcribed RNA of NY99 and NY99-5nt [24]. RNA copies for COAV997-5nt were also determined in samples from COAV997-IC, NY99-IC and CA-04 singly infected groups and reciprocally to rule out cross-reactivity and to extend qRT-PCR validation.

One- and two-way ANOVAs were used for comparison between groups (Graph Pad Prism software, version 5) to determine statistical significance of RNA copies, viral titers and body temperatures between singly and within competitively infected groups (p <0.05). If significant differences were found, a Tukey-Kramer multiple comparison test was used for *post-hoc* analysis between means with α = 0.05.
